# Supplementary material for: PrgE: an OB-fold protein from plasmid pCF10 with striking differences to prototypical bacterial SSBs
Source: Life Sci Alliance. 2024 May 29;7(8):e202402693. doi: 10.26508/lsa.202402693 (PMC11137577; doi:10.26508/lsa.202402693)
Supplement: Supplementary file 1 [file LSA-2024-02693_TableS1.docx]

**Table S1** Structural homology searches using Foldseek reveal low homology to other characterized proteins.

| **Using TM-align mode** | | | | |
| --- | --- | --- | --- | --- |
| **Target (PDB code & chain)** | **Description** | **Species** | **Seq. Id.** | **TM-Score** |
| 3Q6C_O | Duf2500 | *Klebsiella variicola* | 7.3 | 0.488 |
| 5LY5_A | Arcadin-1 | *Pyrobaculum calidifontis* | 8.6 | 0.476 |
| 3RD4_A | PROPEN03304 | *Proteus penneri* | 1.6 | 0.475 |
| 3G48_A | CsaA | *Bacillus anthracis* | 6.6 | 0.474 |
| 7XHS_A | CipA | *Photorhabdus luminescens* | 6.3 | 0.474 |
|  | | | | |
| **Using 3Di/AA mode** | | | | |
| **Target** | **Description** | **Species** | **Seq. Id.** | **E-value** |
| 1XJV_A | POT1 | *Homo sapiens* | 14.9 | 9.12e-3 |
| 6I52_C | RPA | *Saccharomyces cerevisiae* | 10.2 | 4.23e-3 |
| 3KJP_A | Pot1 | *Homo sapiens* | 14 | 1.50e-2 |
| 7R7J_B | RadD | *Escherichia coli* | 8.7 | 1.14e-2 |
| 3U58_D | Teb1 | *Tetrahymena thermophila* | 8.9 | 1.08e-2 |
