# Supplementary figures and images for: PrgE: an OB-fold protein from plasmid pCF10 with striking differences to prototypical bacterial SSBs
Source: Life Sci Alliance. 2024 May 29;7(8):e202402693. doi: 10.26508/lsa.202402693 (PMC11137577; doi:10.26508/lsa.202402693)

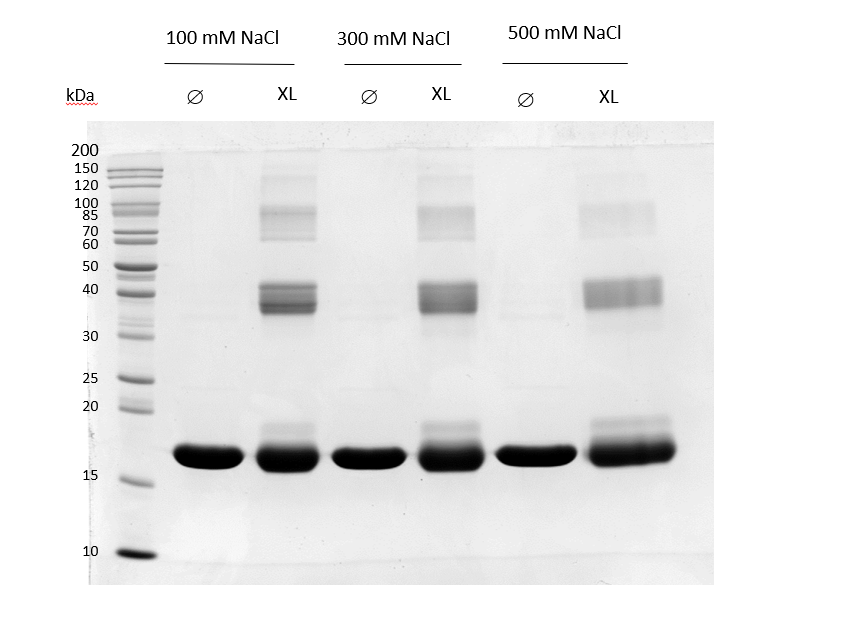

Supplement: Supplementary file 2 [file LSA-2024-02693_SdataF5.tif]

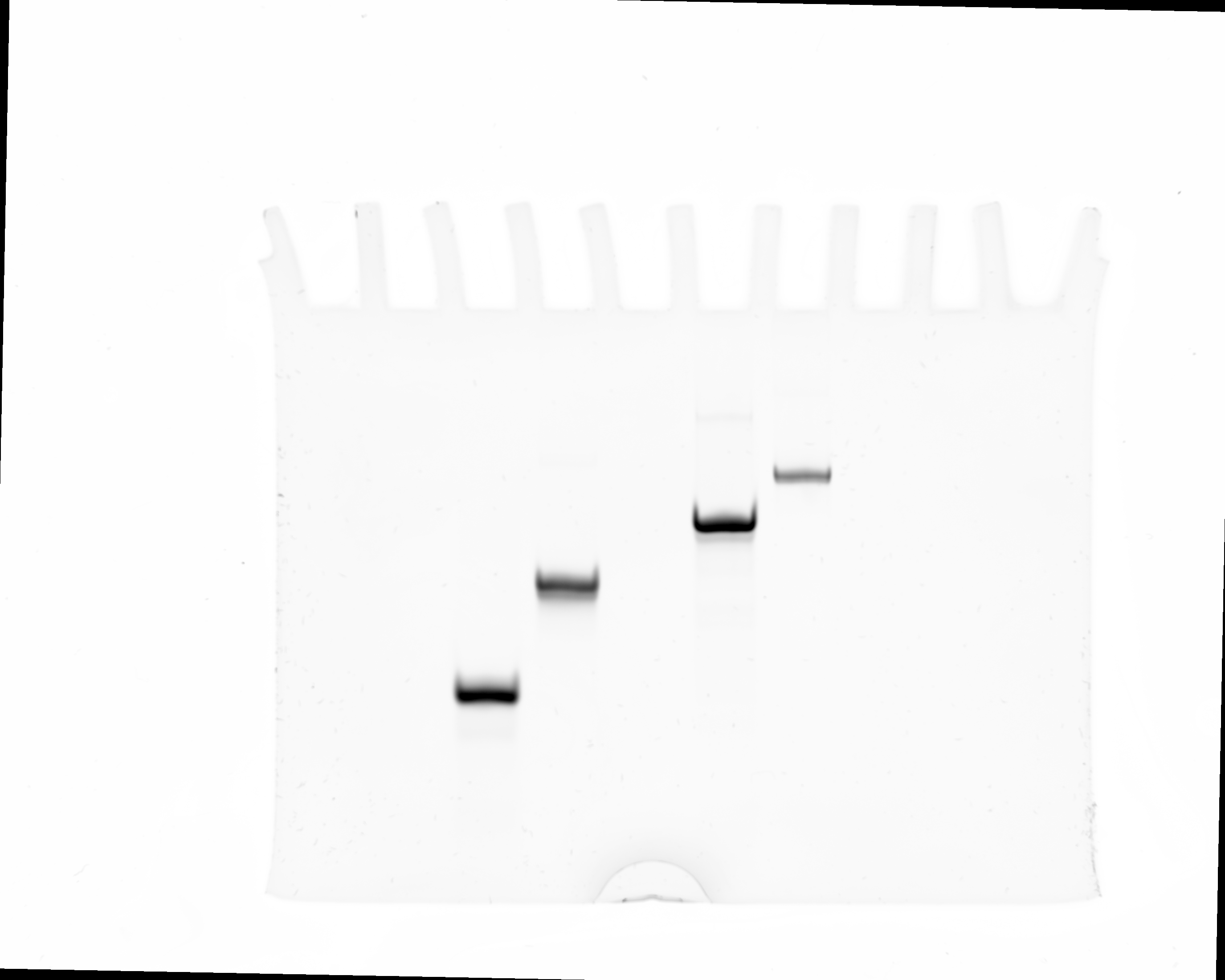

Supplement: Supplementary file 4 [file LSA-2024-02693_SdataFS4.tif]

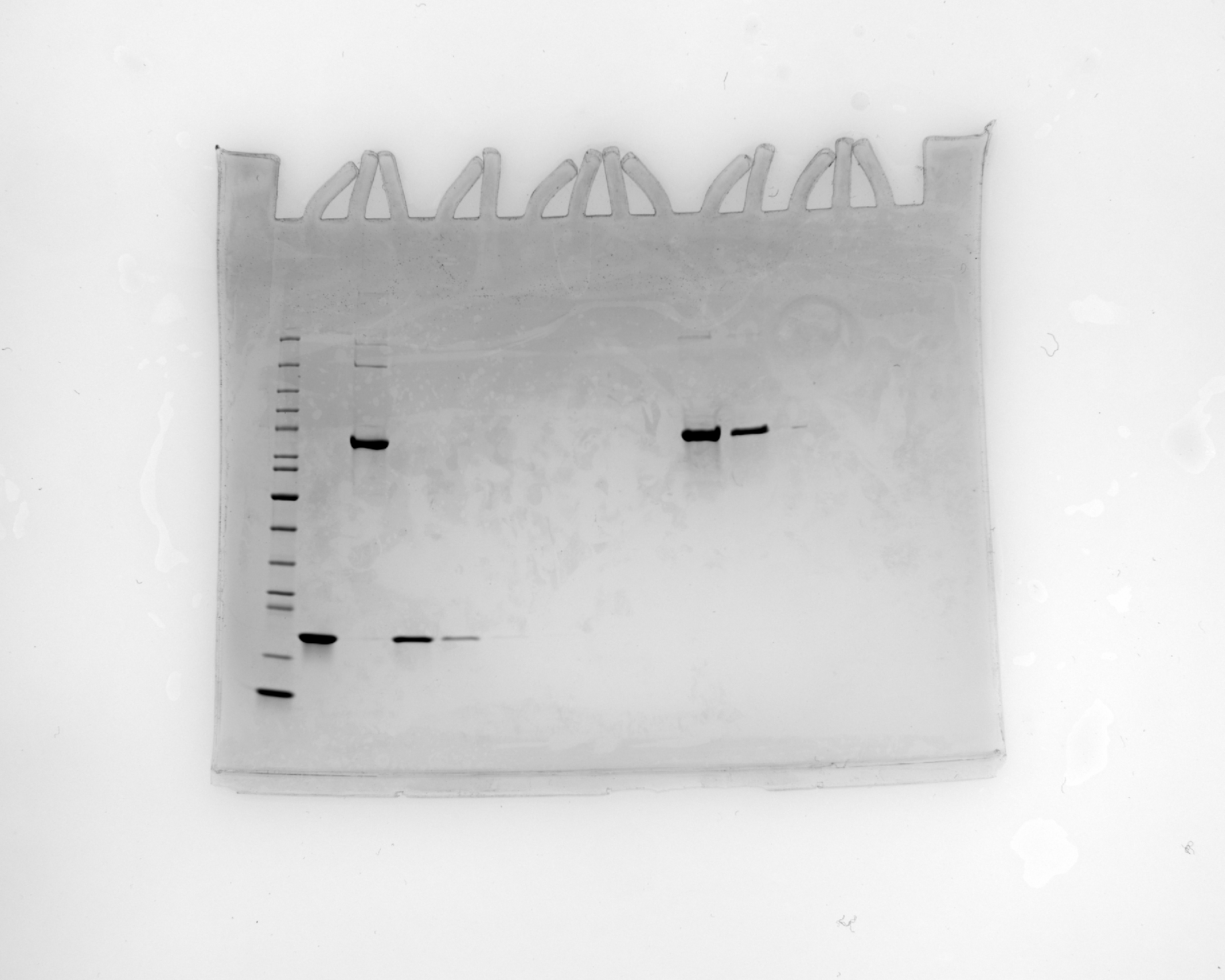

Supplement: Supplementary file 5 [file LSA-2024-02693_SdataF8.1.tif]

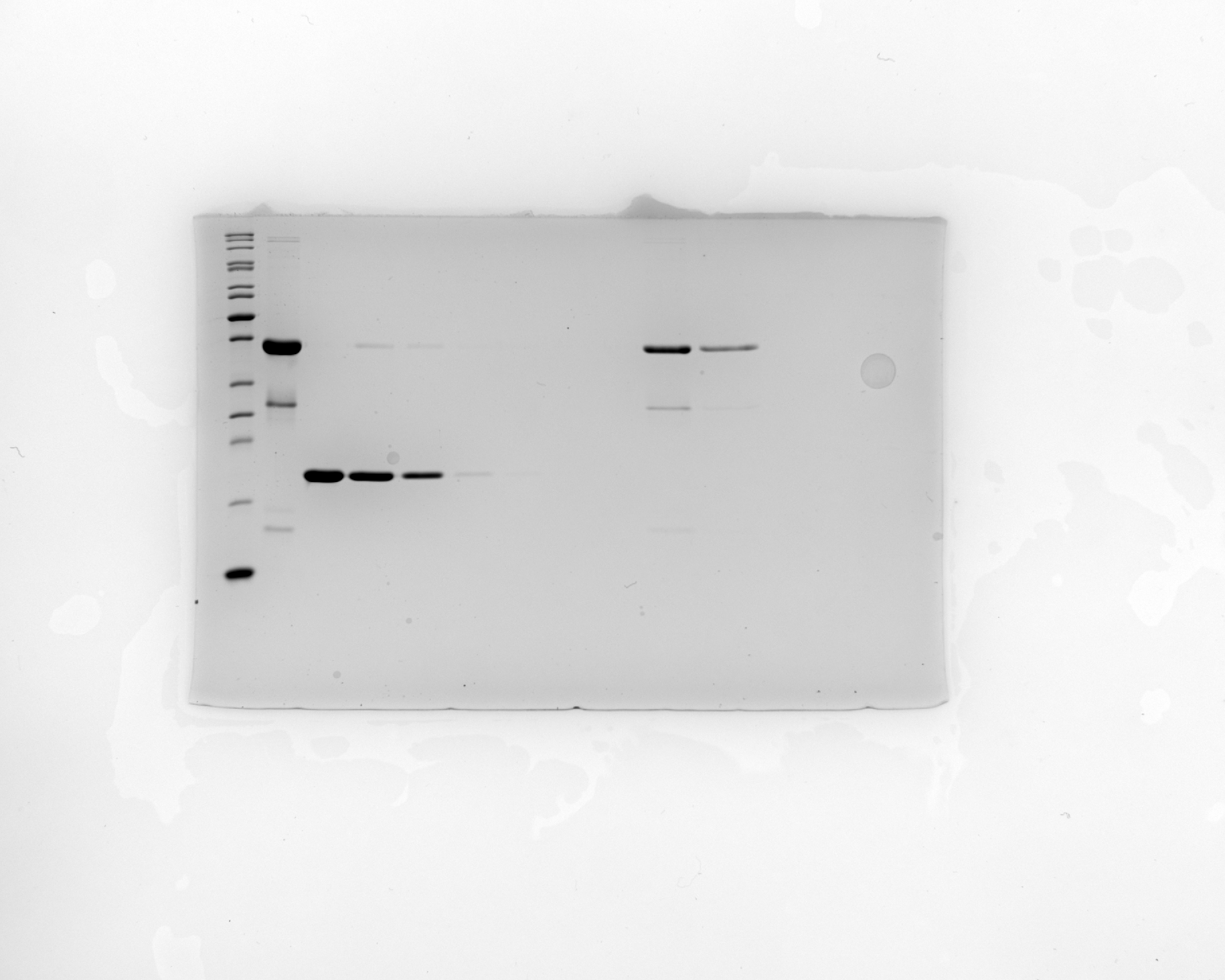

Supplement: Supplementary file 6 [file LSA-2024-02693_SdataF8.2.tif]
