# Supplementary material for: PrgE: an OB-fold protein from plasmid pCF10 with striking differences to prototypical bacterial SSBs
Source: Life Sci Alliance. 2024 May 29;7(8):e202402693. doi: 10.26508/lsa.202402693 (PMC11137577; doi:10.26508/lsa.202402693)
Supplement: Supplementary file 3 [file LSA-2024-02693_TableS2.docx]

**Table S2** Strains, plasmids and oligonucleotides used in this study.

| **Strain, plasmid, or oligonucleotide** | **Relevant features or sequences (5’-3’)** | **References/Manufacturer** |
| --- | --- | --- |
| **Strains (*E. coli*):** | | |
| TOP10 One Shot | Cloning host | Thermo-Fisher |
| ArcticExpress (DE3) | Expression host for PrgE | Agilent Technologies |
| Origami^TM^ (DE3) | Expression host for PcfG | Sigma-Aldrich |
| BL21 (DE3) | Expression host for PcfF | New England Biolabs |
|  | | |
| **Strains (*E. faecalis*):** | | |
| OG1RF | Resistant to fusidic acid | (Dunny et al, 1981) |
| OG1RF:pCF10 | Resistant to fusidic acid and tetracycline | (Dunny et al, 1981) |
| OG1RF:pCF10Δ*prgE* | Resistant to fusidic acid and tetracycline | This study |
| OG1ES | Resistant to erythromycin | (Staddon et al, 2006) |
|  | | |
| **Plasmids:** | | |
| pCJK218 | Allelic exchange plasmid | (Vesić & Kristich, 2013) |
| pCF10 | Pheromone-inducible conjugative plasmid | (Dunny et al, 1981) |
| pINIT_kan | FX cloning intermediate vector |  |
| P7XC3H | FX cloning expression vector with a C-terminal 10-His-tag | (Geertsma & Dutzler, 2011) |
| PrgE-p7XC3H | Vector expressing PrgE with C-terminal 10-His-tag | This study |
| ΔN-PrgE-p7XC3H | Vector expressing PrgE (residues 13-144) with C-terminal 10-His-tag | This study |
| PcfG-pET24d | Vector expressing PcfG with N-terminal 10-His-tag | This study |
| PcfF-pGEX-6P-2 | Vector expressing PcfF with N-terminal GST-tag | (Rehman et al, 2019) |
|  | | |
| **Peptides** | | |
| cCF10 | Sequence LVTLVFV | (Antiporta & Dunny, 2002) |
|  | | |
| **Primers** | | |
| PrgE_FX_F | ATATATGCTCTTCTAGTAAATATGAACGTCCATTAAAAAGAGAG | This study |
| ΔN-PrgE_FX_F | ATATATGCTCTTCTAGTAAGGAGTTTGAGTTAGGCACGCACGCG | This study |
| PrgE_FX_R | TATATAGCTCTTCATGCCCAATCTTCTTCAGTATTGCTTTCTGA | This study |
| PrgE-DF-R | CACACCATGGTCAATGCAATGTTAGTTAATAGCT | This study |
| PrgE-DF-F | CACAGTCGACAGCAATACTGAAGAAGATTGGT | This study |
| PrgE-UF-R | CACAGTCGACACGTTCATATTTCATAGAATTG | This study |
| PrgE-UF-F | CACAGGATCCAATTCTAATTACGTATGAGAT | This study |
| PcfG_F | GTAAAGGTCTCAGGTGGTATGGTGTATACAAAACATTTTGTTATTC | This study |
| PcfG_R | GTAAAGGTCTCAAGCTTATAGTTTGGGCTTAATGTCGG | This study |
|  | | |
| **Oligos** | | |
| 60-mer_F | [FITC]-CAGTGACAGTCTCCACGGTGAAGCAGTCGTACCTCTTGACGCATGAATAGATATATGTTA | Eurofins |
| 60-mer_R | TAACATATATCTATTCATGCGTCAAGAGGTACGACTGCTTCACCGTGGAGACTGTCACTG | Eurofins |
| 30-mer_F | [FITC]-CAGTGACAGTCTCCACGGTGAAGCAGTCGT | Eurofins |
| 30-mer_R | ACGACTGCTTCACCGTGGAGACTGTCACTG | Eurofins |
| Poly-A 60-mer | AAAAAAAAAAAAAAAAAAAAAAAAAAAAAAAAAAAAAAAAAAAAAAAAAAAAAAAAAAAA | Eurofins |
