# Supplementary material for: PrgE: an OB-fold protein from plasmid pCF10 with striking differences to prototypical bacterial SSBs
Source: Life Sci Alliance. 2024 May 29;7(8):e202402693. doi: 10.26508/lsa.202402693 (PMC11137577; doi:10.26508/lsa.202402693)
Supplement: Supplementary file 7 [file LSA-2024-02693_TableS3.docx]

**Table S3** Data collection and refinement statistics.

| **Data collection summary** | **PrgE apo structure** | **PrgE DNA-bound** |
| --- | --- | --- |
| Resolution range | 48.38 – 2.51 (2.56-2.51) | 49.74 - 2.67 (2.77 - 2.67) |
| Space group | P 2_1_ 2_1_ 2_1_ | P 2_1_ 2_1_ 2_1_ |
| Cell dimensions |  |  |
| a, b, c (Å) | 58.175 58.17 87.099 | 75.776 90.043 131.848 |
| α, β, γ (°) | 90 90 90 | 90 90 90 |
| Total reflections | 347213 (18816) | 175730 (17998) |
| Unique reflections | 10486 (537) | 26266 (2536) |
| Multiplicity | 33.1 (35.04) | 6.7 (7.0) |
| Completeness (%) | 99.6 (100) | 99.75 (99.06) |
| Mean I/sigma (I) | 21.4 (2.39) | 10.71 (0.95) |
| R-meas | 13.9 (1.718) | 0.1158 (2.067) |
| CC(1/2) | 0.999 (0.852) | 0.998 (0.628) |
|  |  |  |
| **Refinement summary** |  |  |
| Resolution range | 48.38-2.7 (2.77-2.7) | 49.74 - 2.67 (2.74 - 2.67) |
| R-work | 0.2345 (0.327) | 0.2305 (0.468) |
| R-free | 0.2777 (0.419) | 0.2523 (0.492) |
| Number of non-hydrogen atoms | 2165 | 3716 |
| protein | 2135 | 3372 |
| DNA |  | 315 |
| other ligands | 11 | 20 |
| solvent | 19 | 9 |
| RMS (bonds) | 0.002 | 0.006 |
| RMS (angles) | 0.67 | 1.05 |
| Ramachandran favored (%) | 98.02 | 96.52 |
| Ramachandran allowed (%) | 1.98 | 3.48 |
| Ramachandran outliers (%) | 0.00 | 0.00 |
| Average B-factor | 64.0 | 95.76 |
| protein | 64.29 | 94.37 |
| DNA |  | 111.27 |
| other ligands | 101.09 | 99.49 |
| solvent | 49.62 | 63.43 |

Statistics for the highest-resolution shell are shown in parentheses.
